# Can Machine Learning Predict the Space Group Preference of Organic Molecules?

**DOI:** 10.1021/acs.cgd.5c01648

**Published:** 2026-04-16

**Authors:** Hannah Gittins, Graeme M. Day

**Affiliations:** School of Chemistry and Chemical Engineering, 7423 University of Southampton, Southampton SO17 1BJ, U.K.

## Abstract

Crystal structure prediction (CSP) is a valuable computational technique used to anticipate the likely crystal structures of a compound of interest. These methods have been proven useful in research and development of pharmaceutical solid forms and in guiding the discovery of materials with targeted properties. Despite success of CSP in these areas, its widespread application remains limited by computational cost. One approach to reduce the computational cost of CSP is to limit the search space of generated crystal structures; it is common practice to limit the search to a selection of the most frequently observed space groups, with the associated risk of excluding the space group of an observed crystal structure. As an attempt to reduce computational cost and ambiguity when choosing a set of space groups for CSP, we investigate the use of machine learning models to predict the most likely space group(s) of a given organic molecule. We find that both random forests and graph neural networks provide accuracies far above random, and better than what is achieved by selecting based on the overall space group frequencies observed for organic molecular crystals. The best model, using a graph neural network, achieves a maximum accuracy of 47.2% for single (top-1) space group prediction, which is an improvement of 8.2% above the reference. This model was trained with 3-dimensional molecular information, which improved accuracies compared to a model trained with only 2-dimensional bonding information. Furthermore, we found that random forest models performed best when both chemical and geometric molecular features are included in training, which indicates that both are important in defining a molecule’s preferred space groups.

## Graphical abstract

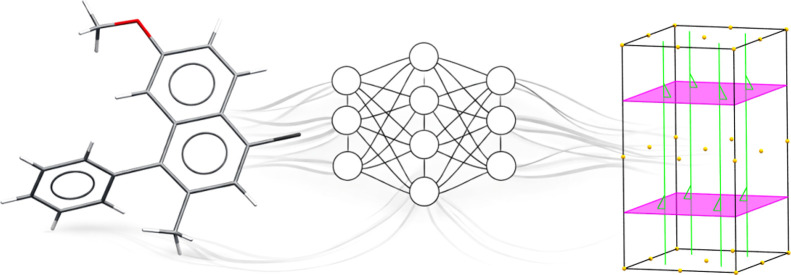

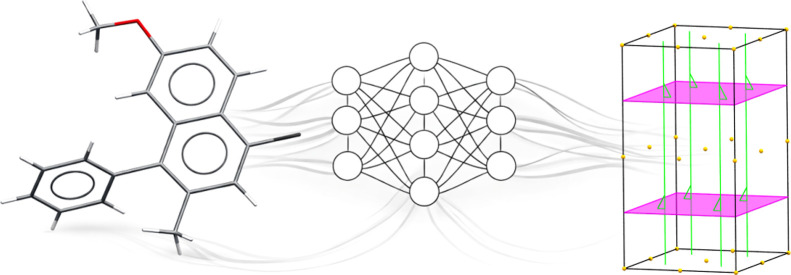

## Introduction

Organic molecules are present in nearly every aspect of our daily lives, from our medications^1^ to the dyes and pigments^2,3^ in our clothes and the food that we eat.^4^ These materials are often formed by organic molecules crystallizing into a solid. The crystal structure determines the physiochemical properties of the material, and a given molecule can have more than one stable structure (polymorph) with different properties. The differences in physiochemical properties can have dramatic consequences: changes in polymorph affect the change transport properties of organic semiconductors,^5^ impact sensitivity of explosives,^6^ adsorption properties of porous solids^7,8^ and dissolution properties of pharmaceuticals.^9^ The ability to predict the likely crystal structures of a molecule can help anticipate polymorphism and the properties associated with a molecule’s crystal structure.

As a result, computational methods for crystal structure prediction (CSP) are valuable in guiding materials discovery and are continually being developed. Search-based CSP can be summarized into a few key steps. First, the 3D coordinates of the molecule(s) are generated and geometry optimized to a neutral starting point (gas-phase) or set of conformations. Then the possible crystal structures are generated, usually within a fixed set of the 230 possible space groups, and these trial structures are lattice energy minimized before removing duplicate structures. The lowest energy predicted structures are assumed to be the most likely to observe experimentally, so these are analyzed in more detail and used as structures from which materials properties can be predicted.

The Cambridge Crystallographic Data Centre (CCDC) holds blind tests to evaluate current CSP methods and encourage development in the field. In the most recent blind test^10,11^ the importance of continued development and innovation was noted, including the need to reduce computational cost and environmental impact of CSP calculations. One possible area to reduce computational cost is to limit the search space of generated crystal structures. It is common to limit the search to a selection of space groups, as seen in the majority of methods from the blind tests. However, limiting the search cannot guarantee that the correct space groups have been chosen.

The frequencies with which organic molecules crystallize into the 230 space groups is very uneven^12^ and large-scale CSP on over 1000 molecules shows that these same space group preferences are found within the low-energy regions of crystal energy landscapes.^13^ The preference for certain space groups is, in part, driven by close packing; Kitaigorodskii demonstrated^14^ that certain combinations of symmetry operators are more effective at promoting close packing of molecules. Further observations have been made that relate specific molecular features to space group symmetry:^12^ for example, the finding that mirror planes are always occupied relates molecular planarity to certain space groups, and inversion centers arrange some self-complementary hydrogen bonding functional groups favorably. Therefore, in an effort to reduce computational cost of CSP by concentrating computational effort on a subset of space groups, in this study we investigate the use of machine learning models to predict the space group of a given organic molecule.

Machine learning and artificial intelligence are rapidly developing fields within chemistry, science and society as a whole. Machine learning of space groups has been attempted for inorganic materials. Liang et al. applied neural networks to predict the space group of a given inorganic material^15^ by first predicting the Bravais lattice, then the space group. All the space group prediction models outperformed what they defined as random accuracy, calculating accuracy by assuming that the Bravais lattice was correctly predicted in the first step, although the Bravais lattice models achieved an accuracy of ∼50–70%, depending on the model used. Therefore, the overall accuracy of the model is significantly lower if the accuracy of the predicted Bravais lattice is taken into account. Other work in the area includes a model trained to predict the space group of 2D inorganic materials using deep neural networks,^16^ random forests to predict the space groups of inorganic compounds^17^ and space group prediction for alloys using neural networks.^18^ During the preparation of this work, Taniguchi and Fukasawa published a CSP method^19^ that makes use of machine learning to direct its structure search. Their method includes a light gradient boosting machine to select space groups from a preselected set of 32 with quoted accuracy above 90% when taking all space groups predicted above a given threshold (10^–2^), which typically includes 5–10 space groups.

Recently, graph neural networks (GNNs) have gained traction with their ability to learn over a graph, allowing them to learn directly from a molecule.^20^ The ability to learn from the molecule directly makes GNNs powerful tools in chemistry, when compared to models where the scientist calculates the features before training. Here, we have applied GNNs to space group prediction and compared their performance to random forest (RF) models, which have also been applied to chemistry applications.^17,21^ RFs are made up of a collection decision trees, and each decision tree is trained to learn the mapping between the input (molecular features) and output (space group) using a greedy algorithm. As the RF uses a greedy algorithm, it is less computationally demanding than NNs. Therefore, RFs are a good first pass at applying machine learning to space group prediction.

Unlike GNNs, the features used in RFs must be calculated before training. The geometry and chemical environment of a molecule have a large influence on the crystal structure and therefore the space group. For example, a space group including a mirror plane would struggle to optimally pack a molecule with a bump and hollow; space groups excluding this operation would be better suited to maximize packing efficiency. On the other hand, the formation of hydrogen bonds can be more energetically important than close packing, such that the absence or presence of hydrogen bond acceptors and donors can influence the favorable crystal structure and therefore space group. As a result, geometric and chemical features are both potentially useful features to learn this mapping and are included in our training of RFs for organic molecular space group prediction.

## Method

### Data Wrangling

The data set was collected from the Cambridge structural database (CSD), version June 2023.^22^ Using the CSD ConQuest software package, the search was limited to return structure reference codes that were constrained to contain only organic molecules, *Z*′ = 1, one residue (i.e., one type of molecule in the structure), no errors, no ions, no polymers and no organometallics (as the CSD defines it). Crystal structures where the asymmetric unit consists of multiple part-molecules, (e.g., *Z*′ = 0.5 + 0.5) were excluded.

To handle polymorphism, where a molecule is observed to crystallize in more than one crystal structure, we created two versions of the data set. A first version of the data set retained only one polymorph (and, thus, one space group) per molecule, for which we retained the first structure listed in the CSD, assuming that this corresponds to the first observed crystal structure of that molecule. A second version of the data set, the “All polymorphs (with unique space groups)” data set, retained all polymorphs with unique space groups. 86 space groups are represented in the data set.

Any crystal structures that contained errors were also removed at this stage. The molecular geometry was extracted from each crystal structure and the molecular geometries were optimized using the ORCA software^23^ with the low cost B97-3c density functional method.^24^ B97-3c was chosen as it is relatively low cost and has been shown to perform similarly to more expensive methods.^25^ This left a final data set of a size of 224,634 molecules (without polymorphs) and 224,842 entries (with polymorphs). A data set was also kept with molecular geometries taken directly from the crystal structures without optimization; this set, which excludes polymorphs, had 224,668 molecules (the number differs from the set with optimized geometries, in which errors during optimization caused some molecules to be excluded).

Balanced data sets were also created in which the number of molecules with each space group in the set was equal. The balanced data sets were subsets of the original, larger data set. One balanced data set was limited to the 25 most frequently observed space groups in the full data set, the size of the data set being limited to the least frequently observed space group within this group. (The 25 most frequently observed space groups cover 99.03% of *Z*′ = 1 crystal structures of organic molecules from the CSD. This balanced data set size was 6350 molecules, selected randomly from the full data set. A second balanced data set was created that was limited to only the top 10 most frequently observed space groups; this balanced data set’s size was 19,950 molecules.

Each data sets was split into a training, validation and test set. A 95/2.5/2.5% split was used for the unbalanced (original) data set, and the balanced data set was split as roughly 80/10/10% (to keep the space groups even across training, test, and validation set, the split ended up being closer to 80.2/9.9/9.9% for the smallest, 25 space group, data set).

### Random Forest

#### Feature Generation

The geometry and chemical features, such as the absence or presence of hydrogen-bond donors and acceptors, are expected to influence the crystal structure and therefore the space group that a molecule adopts. Therefore, we calculated the features that describe these attributes, which we summarize as geometric, general, and chemical features. Geometric and general molecular features were calculated using the RDKit^26^ library and the CCDC python api.^27^ The general features include: molecular weight, number of atoms, number of rings and number of aromatic rings. The geometric features include: molecular asphericity, eccentricity, normalized ratios of the principle moments of inertia and mean atomic displacement from the plane of best fit (as a measure of the planarity of the molecule), as well as information on the molecular symmetry: the number of symmetry operations and a count of each type of symmetry operator. (The full set of features is listed in the Supporting Information.) These features are intended to capture the geometric information on the molecule. To test the influence of the molecular geometry on space group prediction, geometric features were calculated from the molecular geometry as reported in each crystal structure, and also with the optimized (B97-3c) molecular geometries. A small number of molecules failed geometry optimization, so were discarded from the corresponding data set.

Chemical features were generated from the SMILES strings using RDKit. In this case, the chemical features were the count of each of 18 common functional groups in the molecule. This includes: alcohol, amide, imine, nitro, nitrile, amine, ether, aldehyde, halide, ketone, carboxylic acid, anhydride, ester, thiol, thiocarbonyl, thioether, sulfone and phosphoric acid. Topological polar surface area was also calculated and included with these features, as this gives a measure of the polarity of the molecule.

Chiral molecules crystallized from stereochemically pure samples are limited in their space group choice to Sohncke space groups. Unfortunately, while the chirality of molecules is available, we do not have the information for each chiral molecule of whether it has been crystallized from a pure or racemic mixture. This information could be useful for future model development if the relevant information could be mined from the relevant publications. We experimented with training separate models for chiral and nonchiral molecules, but removing chiral molecules from the overall data set was found to reduce the space group prediction accuracy for nonchiral molecules. So, apart from including point group operations as features, chirality is not explicitly included as a feature. However, the user can select only relevant space groups when using the models, if they are making predictions for chiral molecules.

#### Model

Before training the random forest models, the hyperparameters were tuned for optimal performance (using Optuna^28^) on the balanced data set (before removal of irrelevant features). It was too computationally demanding to hyperparameter-tune with the full data set, so we assume that the hyperparameters that best suit the balanced set would also suit the unbalanced set. Furthermore, the data set was assessed to remove irrelevant features: features that had high correlation with another feature were removed (and one feature retained) and features that had low variance across the data set were also removed. Again, feature removal was performed on the balanced data set and the same features were removed for the unbalanced data set. (More details are provided in the Supporting Information).

Three random forest models were trained using all features for the full (unbalanced) data set. For the data set where one polymorph (so one space group) was retained per molecule, we trained a model (RF_A) where geometric features were calculated from the optimized molecular geometries and another model (RF_B) using the molecular geometries taken directly from the crystal structures. A third model (RF_C), trained using all polymorphs with unique space groups for each molecule, used the geometric features calculated from optimized molecular geometries. Two further models were trained in the same way as RF_A, but with subsets of the molecular features: one with general and geometric features only, and another with chemical features only. The models were trained using the premade random forest available in the scikit-learn^29^ python library.

### GNN

#### Feature Generation

For a GNN, rather than generating or calculating features, a molecular graph is made. These molecular graphs were made using PyTorch,^30^ PyTorch geometric^31^ and RDKit. The bonds/edges were assigned the following attributes: bond type/order, conjugation type and ring value (whether the bond is in a ring or not). For atoms/nodes: atomic number, hybridization of the atoms, formal charge of the atom, aromaticity and the calculated Gasteiger partial charge (calculated using RDKit). The atomic coordinates were also included as node attributes in some models.

#### Models

All the GNNs were created and trained using PyTorch and PyTorch geometric. Two main models were evaluated: NNConv, which is a graph convolutional network with a neural network over the edge features, and an equivariant graph neural network (EGNN), see Table 1.

**Table 1:** Overview of Graph Neural Network Models Applied to Space Group Prediction

model	description	reference(s)
NNConv	graph convolutional network involving the edge features. The edge features go through a neural network before being used to influence the “messages” passed between the nodes	Gilmer et al.^32^ Simonovsky et al.^33^
EGNN	a graph neural network with equivariant graph layers. This retains the 3D geometry without requiring fixed Cartesian coordinates	Satorras et al.^34^

Full hyperparameter tuning was not performed on the GNNs, as this was found to be too computationally demanding. Through trial and error, the models performed best (or the loss responded best) when there were 3 message passing layers followed by a global mean pool (this pooled the node embedding into one graph embedding) followed by 2 fully connected neural network layers. For the NNConv, an edge NN is included; we found the model performed best with only 1 NN layer on the edges, any more leads to overfitting. Furthermore, the addition of graph normalization^35^ and gradient clipping (to prevent exploding gradients) improved the loss of the models. The optimizer, activation, and loss calculators were not explored manually but set to the Adam optimizer,^36^ RELU activation function (and Softmax for the last layer) and cross-entropy loss, as these are commonly used.

Four GNN models (see Table 2) were trained on the full (unbalanced) data set. GNN_1 does not use atomic coordinates; instead, it uses the connectivity and bonding of the molecule, but not its exact geometry. GNN_2 includes atomic coordinates, but is not invariant to rotation or translation of the molecule. GNN_3 addresses the invariance by data augmentation: the model was trained with several versions of each molecular geometry. These were obtained by performing a random rotation about each of the *x*-, *y*- and *z*-axes (and retaining the original coordinates), storing the coordinates after rotation in the molecular graphs. This data augmentation was applied at the batch level, meaning that a batch size specified as 16 on the input became 64 (16 × 4) after augmenting the data and was sent through the model for training. We found that this model responded best with an increase in model complexity to handle the increased data set size and complexity of data augmentation. Therefore, we included more layers. GNN_4 used equivariant graph layers, so that the 3D molecular geometry could be retained without requiring fixed Cartesian coordinates for the atoms.

**Table 2:** Summary of the Trained GNN Models, Where MP = Message Passing Layers or Equivalent, EL = Edge Layer and NN = Neural Network Layer

model	MP layer	layers
GNN_1 (no coordinates)	NNConv	3MP (1EL), 2NN
GNN_2 (3D coordinates)	NNConv	3MP (1EL), 2NN
GNN_3 (augmented data)	NNConv	4MP (2EL), 3NN
GNN_4 (equivariant)	EGNN	3MP, 2NN

We initially trained all models for a maximum of 500 epochs; however, models 1 and 2 did not appear to have converged, so they were trained for a further 500 epochs (a total of 1000 epochs), see Section 2.1. The models with the best validation loss were retained and used for testing.

### Model Evaluation

For all models, the results are evaluated by the accuracy of predictions on the held-out test set molecules. In the case of models trained on the balanced data set, we assess the accuracy against an unbiased, random selection of space groups for each molecule. For models trained on the full, unbalanced data set, we set a more stringent reference, which is the accuracy that would be obtained by assigning space groups randomly, but with a probability based on the observed distribution of space group frequencies.

As CSP is rarely performed in a single space group, we evaluate the top-*N* accuracies, for *N* = 1, 3, 5, and 10. For top-1, we take the space group that is predicted by most trees in the random forest (or highest probability from the GNN Sofmax output layer) and for *N* > 1, the *N* most likely space groups are taken from the random forest model as the *N* most commonly predicted from the trees (or *N* highest output GNN nodes).

The reference accuracy for the unbalanced data set was calculated by assuming the *N* most frequent space groups (for top-*N* accuracy) by frequency order in the CSD; the accuracy is the proportion of the relevant test set that represented by these *N* space groups. For balanced data sets, the random accuracy was calculated as 100/*M*, where *M* is the number of space groups in the data set.

For the random forest model we also assessed the prediction precision and recall of the models.

1 $$recall=\frac{tp}{tp+fn}$$

2 $$precision=\frac{tp}{tp+fp}$$

where tp, fp and fn are the true positives, false positives and false negatives, respectively, of predictions for the test set.

To calculate the precision, the frequency of the correctly predicted class, and recall, the frequency with which the model recalled the correct class, when predicting Top *N* space groups, we assumed the following.

- If the correct space group is in the Top N predicted space groups (from the model), then the true positive, tp, is 1 (for the singular example).
- By that logic, if the correct space group is not in the Top *N* predicted space groups, the false negative, fn, is assigned 1 (for that singular example).
- It was assumed that every space group assigned incorrectly is a false positive, fp. However, without normalizing it would cause scaling issues and cause the fp score to explode (and the precision and recall to shrink), so fp was scaled by 1/*N*.

The tp, fp and fn were calculated for each space group and the recall (1) and precision (2) were calculated for the 1, 3, 5, and 10 top space groups.

## Results & Discussion

### Random Forest

#### Statistically Distributed Data Set

The data sets were divided into a training, validation, and holdout test set (see Section). The accuracy of the model on the test set (Table 3) is evaluated for the top-1, 3, 5, and 10 predicted space groups, as CSP methods usually assume a set of *N* likely space groups when generating trial crystal structures.

**Table 3:** Accuracy for All Three Random Forest Models Trained on the Optimized Geometries, Non-optimized Geometries and All Polymorphs (with Unique Space Groups) Included Unbalanced Datasets^a^

	RF_A: opt geometries			RF_B: non-opt geometries			RF_C: all polymorphs included		
	ref./%	acc./%	Δ/%	ref./%	acc./%	Δ/%	ref./%	acc./%	Δ/%
top 1	38.96	41.93	2.97	40.22	44.58	4.36	38.57	43.39	4.82
top 3	73.06	75.66	2.60	73.60	77.51	3.91	72.66	75.97	3.31
top 5	87.09	87.75	0.66	87.11	88.43	1.32	86.60	87.65	1.05
top 10	95.76	96.26	0.50	95.50	95.98	0.48	95.61	94.95	–0.66

a Ref. = accuracy if using the already known statistical distribution of space groups on the respective test set. Δ is the accuracy difference between the random forest model and the reference.

We calculate a reference accuracy for each model (the “accuracy from known statistical distribution”, Table 3) as the accuracy that is returned if we assume that the top-*N* space groups from the CSD distribution for each molecule in the respective test set. For example, the top-1 “accuracy from known statistical distribution” is the accuracy if we assume space group number 14 (*P*2_1_/*c*, the most common space group in the CSD) for every input in the test set (or the frequency of that space group in the test set). The space group prediction models could be useful for guiding CSP space group selection if they outperform this reference.

Model RF_A, which uses the DFT optimized molecular geometries when calculating geometric descriptors, as would be the case in CSP applications, achieves a top-1 accuracy of 41.93%. This is much better than random space group prediction among the 86 space groups present in the training data and is approximately 3% higher than the reference. The absolute accuracy might seem low, but should be interpreted in the context of what is known about molecular crystallization: many molecules are polymorphic, sometimes with polymorphs in different space groups. Thus, a high accuracy in predicting a single space group is unrealistic. Furthermore, computational CSP studies show that low energy crystal structures, again often with different space groups, are usually separated by small energy differences; this reinforces the expectation that there is not a one-to-one mapping between molecule and space group. Instead, what we want is to be able to select the subset of most likely space groups: top-3, −5 and −10 accuracies for RF_A are 75.66%, 87.75% and 96.26%.

These top-*N* (*N* > 1) predictions for RF_A outperform the reference, but with a decreasing gap, such that the top-5 and top-10 accuracies from the random forest give only marginally higher accuracy than simply choosing the 5 or 10 most frequently observed space groups. This decreasing advantage over the reference is understandable: the reference accuracy for top-10 prediction is 95.76%, so there is little room for improvement on this by the RF model.

As the top-1 accuracy of model RF_A is only ∼3% above the reference of using already known space group frequencies, we performed 10-fold cross-validation to confirm the significance of the model accuracies (Figure 1). The training and test sets were split 90:10% in the cross-validation process. As the standard deviation in accuracy is much smaller than the difference in accuracies between the RF_A and reference, we are confident that the higher accuracy of the random forest model is statistically significant. These results indicate that the random forest model is learning true relationships between the molecular features and the space group that a molecule adopts.

**Figure 1:**
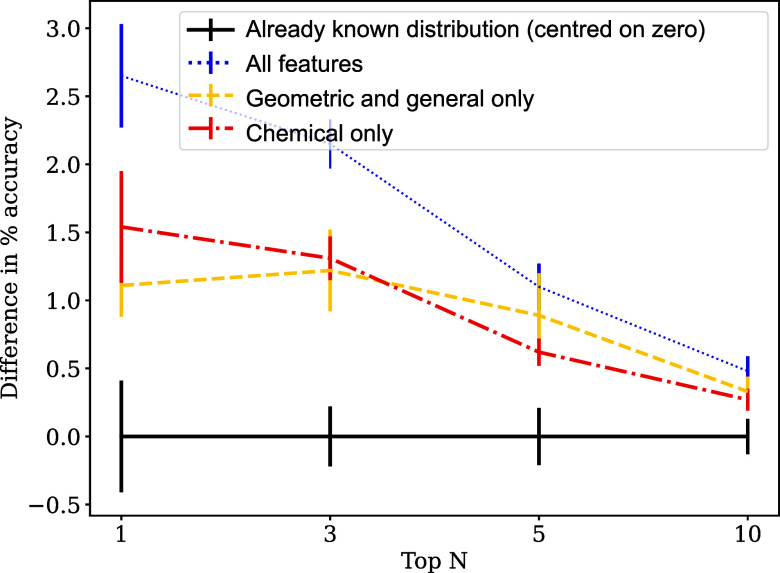
RF_A: 10-fold cross-validation for the random forest model trained on the full (unbalanced) data set. The accuracy of predicting based on the known statistical distribution of space groups is centered at zero. Error bars correspond to 1 standard deviation from cross-validation.

Ablation studies were performed to understand the role of geometric and chemical features: models were trained (with cross-validation) on only the geometric (and general) features and only on the chemical features. The accuracies of these models are both below the RF_A model trained on all features, but above the reference (Figure 1), demonstrating that a combination of geometric and chemical features is used by the random forest in predicting the most likely space groups, and that both classes of descriptors have similar influence on prediction accuracy.

To further investigate the role of geometric features, we compared accuracies of model RF_A to model RF_B, which was trained with the molecular geometries taken directly from the crystal structures (without optimization). Crystal structure prediction studies have shown that the energetic ranking of crystal structures,^37^ and therefore the preferred space group, is strongly influenced by small changes in the geometry of the molecule. Therefore, we expected that a model (RF_B) trained using geometric features calculated directly from the molecular geometry in the crystal structure should perform better than when using DFT optimized molecular geometries (as in RF_A), which lack the influence of crystal packing on the molecular geometry. This effect is borne out in the results (Table 3 and Figure 1S): the RF_B model accuracies are higher than RF_A for top-1 to top-5 space group predictions. The two models perform equally at top-10 space group predictions. These results confirm that geometric features are having a role in the random forest prediction accuracy. Crystal structure prediction studies would not have access to the molecular geometry from the crystal structure, so the slightly lower accuracies obtained with RF_A are the more relevant results for applications in CSP.

The effect of including multiple polymorphs with different space groups during training is tested in model RF_C. The accuracy is improved over RF_A, which only included one space group per molecule, for top-1 to top-5, indicating that the extra information helps with predictions. Top-1 predictions outperform the reference by nearly 5% with this model. However, the RF_C accuracies drop below the reference for top-10 space group predictions and perform poorly when either geometric or chemical features are removed (Figure 2S).

**Figure 2:**
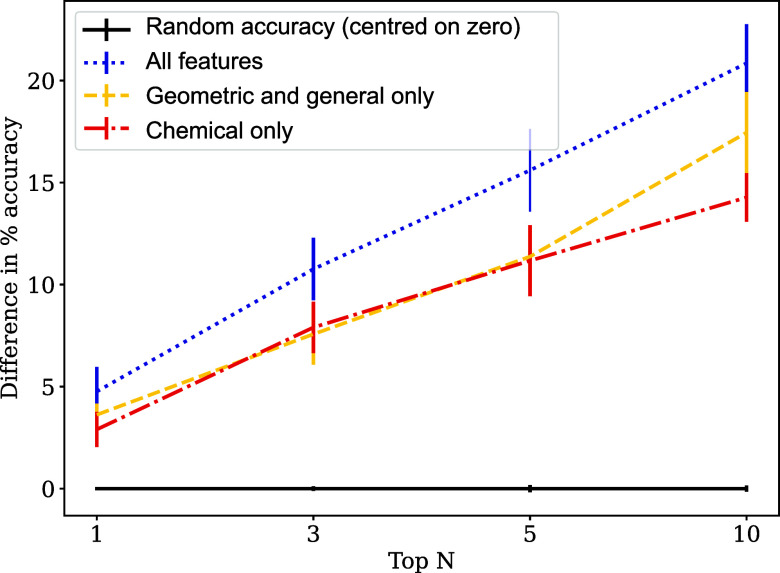
10-fold cross-validation for the balanced models trained on the 25-space group balanced model, to 1 standard deviation. The accuracy of random space group prediction is centered at zero.

#### Balanced Data Set

To investigate the random forest models’ propensity to learn from the biased distribution of space groups in the training set, we show here models trained on balanced training sets and tested with balanced test sets (Table 4 and Figure 2). Geometric features calculated from optimized molecular geometries were used and polymorphs were excluded, so this model should be compared to RF_A. The reference accuracy in this case is the accuracy of predicting a space group randomly; the reference accuracy from the known statistical distribution of space groups no longer applies, as the statistical bias has been removed from the training and test data sets.

**Table 4:** Accuracy for the Random Forest Model Trained and Tested on Balanced Datasets, Using Geometric Features from Optimized Molecular Geometries, and Not Including Multiple Space Groups for Polymorphic Molecules^a^

balanced model (25 space groups)			
	Random accuracy /%	Accuracy /%	Δ /%
Top 1	4	10.08	6.08
Top 3	12	23.68	11.69
Top 5	20	34.72	14.74
Top 10	40	62.24	22.19
balanced model (10 space groups)			
Top 1	10	18.69	8.71
Top 3	30	47.86	17.89
Top 5	50	67.63	17.67

a All (general, geometric and chemical) features were included in the training. The random accuracy was calculated as 100/*M* %, where *M* is the number of space groups represented in the dataset.

We first trained a model on the 25-space group balanced data set. Although the absolute accuracy of the model trained on balanced data is much lower than those trained on the full data set, the model performs better than random, even performing ∼22% better for top-10 space group predictions. The lower accuracy is undoubtedly due, in part, to the much smaller training set size; the balanced set is <3% of the size of the full data set. Unlike when using the full data set, the gain in top-*N* accuracy over the reference improves with increasing *N* (noting that the reference here is different from the unbalanced model).

Although the low overall accuracy of the balanced model means that it is not a useful model, the results demonstrate that the random forest models are not only learning to reproduce the statistical distribution of space groups in the training set, but learn true, predictive relationships between the supplied molecular features and preferred space groups. By removing the statistical bias in the data, the larger improvements relative to the reference suggest that the model learns more effectively from the molecular features. However, the necessarily smaller training set, which is limited by the number of molecules with the least frequently observed space groups, limits this model’s performance.

Table 5 shows the accuracy of the balanced model when tested on a test data set that has a similar space group distribution to the CSD (but limited to the most frequent 25 space groups). The Δ reported in Table 5 compares to the reference accuracy of choosing space groups according to the known frequency distribution, emphasizing that the balanced model is not competitive.

**Table 5:** Accuracy of the Balanced Model When Tested with a Held-Out Dataset That has a Similar Distribution of Space Groups to the CSD (Including Only the Top 25 Space Groups)

	accuracy from known statistical distribution/%	accuracy/%	Δ/%
top 1	38.96	6.90	–32.06
top 3	73.06	18.60	–54.46
top 5	87.09	31.20	–55.89
top 10	95.76	58.90	–36.86

To investigate whether more useful models could be trained with larger balanced data sets, by reducing the number of space groups included in the training, we trained a model on a balanced data set with only the 10 most commonly observed space groups. The balanced data set is over 3× larger than the 25-space group balanced data set, but still less than 9% of the full, unbalanced data set. The results (Table 4 and Figure 3S) show improved top-1, top-3 and top-5 accuracies, and greater accuracy increments above random accuracy, than the smaller balanced set. These results reinforce the finding that the random forest learns meaningful relationships between molecular features and space groups. However, the accuracies remain uncompetitive with those obtained with the models trained on the large, unbalanced data set (Table 3). Reducing the balanced data set to fewer than 10 space groups to further increase the size would not be useful.

**Figure 3:**
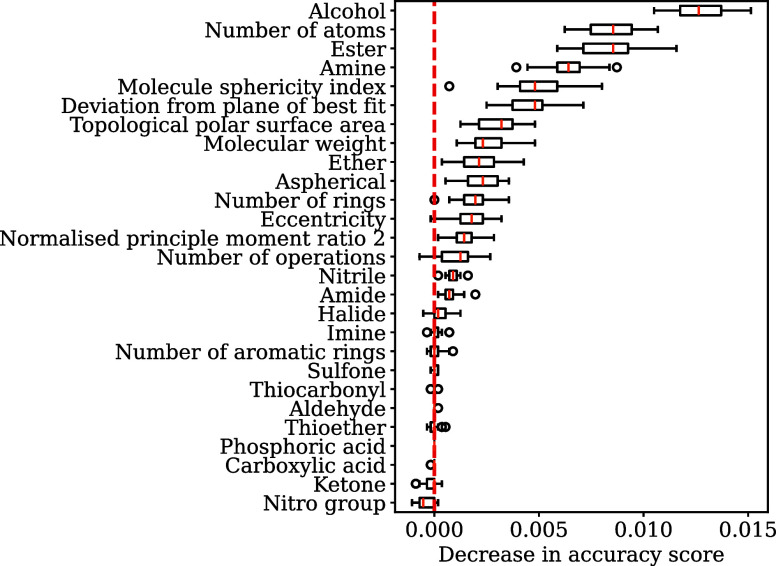
Feature importance for the random forest trained on the unbalanced test set. Features have high importance if they reduce the accuracy when removed from the data set.

#### Feature Importance

From the results presented above, we are confident that the RF model is able to learn useful information on space group preferences from molecular features. Here, we investigate which individual features, or classes of features, are important in making these predictions. Both cross-validation plots (Figures 1 and 2) show that the RF models including all features perform best, followed by models trained using geometric only and chemical only features. This is what we expect, as both the molecular shape and the specific atoms and functional groups play an important role in determining the stable crystal structure for a molecule, and therefore influence the space group. Following on from the cross-validation plots, we also assessed the feature importance for the model using sci-kit learn’s permutation_importance module. (For permutation feature importance, each feature is assessed one at a time, and the values are shuffled. The model’s performance is then assessed with the feature scrambled to see how much of a negative or positive impact that has on the model. A decrease in accuracy indicates that the feature has a large influence on the model’s performance.)

The RF_A unbalanced model attributes the greatest feature importance (Figure 3) to the counts of certain functional groups (alcohol, ester, amine), as well as the number of atoms and measures of the molecular shape: deviations from the plane of best fit, and molecular sphericity. The importance of potential hydrogen bonding groups is unsurprising, as these can have preferred geometries that favor certain symmetry operators. The low importance of carboxylic acids is, however, counterintuitive, as these are known to often form hydrogen bonded dimers around inversion centers.^38^

Some of these features also have high importance for the balanced model, while some of the chemical features (e.g., count of alcohol and ester groups) drop to having no apparent importance (Figure 4S). The balanced model feature importance is less reliable due to the much smaller sampling size of features, as a result of the smaller data set size.

These results give some idea of what is contributing to the model learning the mapping from molecular features to space group. However, the feature importance does not capture how the features work together.

### GNN

The random forest results have shown that relationships can be learned to map a molecule’s features to space group preferences, yet the RF models give small accuracy improvements over the use of the known, uneven space group distribution for organic molecular crystals.

We theorized that either the molecular features used were not complex enough or that the mapping between the molecules and space groups could not be learned effectively by the random forest models. To explore a more complex machine learning model, we experimented with training graph neural networks (GNNs) using molecular graphs as the features. Four models were tested (Table 6), differing in how the molecular geometry is treated.

**Table 6:** Comparison of the GNN Model Accuracies^a^

		GNN_1		GNN_2		GNN_3		GNN_4	
	reference/%	Acc./%	Δ/%	Acc./%	Δ/%	Acc./%	Δ/%	Acc./%	Δ/%
top 1	38.96	43.95	4.99	47.20	8.24	41.10	2.14	37.50	–1.46
top 3	73.06	77.48	4.42	79.86	6.80	73.40	0.64	71.42	–1.64
top 5	87.09	88.35	1.26	90.15	3.06	86.77	–0.32	83.40	–3.69
top 10	95.76	96.31	0.55	96.65	0.89	95.78	0.02	95.57	–0.19

a GNN_1: NNConv with no atomic coordinates (2D molecular graph only). GNN_2: NNConv with atomic coordinates as node features. GNN_3: NNConv trained with augmented data (atomic coordinates afer molecular rotation). GNN_4: EGNN. Reference = accuracy from known statistical distribution (CSD).

Initially, we trained a GNN model (GNN_1) with no 3-dimensional information (ie. no atomic coordinates), where the molecular graph only contains 2-dimensional information on the bonding within the molecules. The model performed surprisingly well (Table 6), with higher accuracies from top-1 to top-10 space group predictions compared to RF_A. As with the random forest models, the improvement over the reference accuracies (predictions based on the known space group frequency distribution) is highest for top-1 prediction, decreasing as the number of predicted space groups increases. As with the random forests, the accuracy for top-10 prediction is only marginally higher than the reference.

In the second model, GNN_2, we introduce the 3D molecular geometry by adding the atomic coordinates as node features. Including the atomic coordinates increases the accuracy relative to GNN_1 for all top-*N*, with an improvement of over 3% for top-1 space group predictions. GNN_2 is particularly impressive when predicting small numbers of space groups: top-1 and top-3 accuracies are 8.2% and 6.8% above the reference (predicting from the known statistical distribution of space groups).

While including atomic coordinates improves the accuracy of the GNN, this model is not invariant to changes in the 3D coordinates from molecular translation or rotation, which could affect how well the model generalizes. To address this issue, we also trained a model (GNN_3) in which the training data has been augmented: each molecule was included 4 times, including atomic coordinates after applying molecular rotations for the 3 added copies. This augmentation effectively quadrupled the training set size and provides the model with the opportunity to learn the invariance to molecular rotation. The GNN_3 model was tested with the original test set (not augmented).

Due to the increase in data set size and variation in the data, GNN_3 struggled to train, and we found it performed best with a more complex model. Therefore, GNN_3 is larger than all the other models (Table 2). Surprisingly, the data augmentation led to a decrease in accuracy (compared to GNN_2) and, apart from top-1 space group prediction accuracy, the accuracy of GNN_3 is very close to or worse than predicting space group based simply on the known space group distribution. The reason for the reduction in accuracy from GNN_2 to GNN_3 is unclear, but could result from data augmentation making it harder for the model to form a clear mapping from molecule to space group.

Finally, an equivariant GNN (GNN_4) was trained but, surprisingly, this model performed worse than all other models, as well as giving worse accuracy than the reference (known statistical distribution of space groups). It is unclear why GNN_4 performed this poorly.

Each of GNN_1, GNN_2 and GNN_3 are an improvement over the reference, demonstrating that the molecular graph provides useful information relating molecular structure to the space group adopted upon crystallization.

## Conclusions

This work has explored whether machine learning methods can predict the likely space group(s) that an organic molecule will adopt. A key motivation for this study is for applications to crystal structure prediction, where limiting which space groups are considered reduces computational cost and time needed for completing predictions.

To begin with, we explored the task of predicting a set of space groups with a random forest model by training the model with geometric, general and chemical features of nearly 225,000 molecules with known crystal structures covering 86 space groups. The random forest models perform much better than random, with top-1 accuracies of 41.9 to 44.6%, top-3 accuracies of 75.7 to 77.5% and top-5 accuracies of 87.7 to 88.4%. While these accuracies are also higher than basing predicting on the overall space group frequencies in the data set, the improvement is small: 3 to 4.8% for top-1 predictions, 2.6 to 3.9% for top-3 prediction, 0.7 to 1.3% for top-5. To investigate whether the random forest models were simply learning the space group frequencies, we trained with chemical or geometric molecular features removed, demonstrating that both types of molecular features are used in making predictions. Further tests were performed, training models on balanced, but much smaller training sets. This models performed significantly better than random (by ∼6% for top-1, ∼12% for top-3 and ∼15% for top-5 for a 25-space group model), confirming that random forest models are able to form a predictive mapping between molecular features and likely space groups.

To investigate whether the features and/or random forest model were not complex enough to make the most predictive mapping, four graph neural network models were also trained, either with only 2-dimensional bonding information (GNN_1) or with the 3-dimensional molecular geometry (GNN_2, 3 and 4). The best of these models, GNN_1 and GNN_2, give higher accuracy than the random forest models with accuracies up to 47.2% (top-1), 79.9% (top-3) and 90.1% (top-5). While the 3-dimensional molecular information in the most successful model, GNN_2, is not invariant to molecular rotation or translation, attempts at using data augmentation or equivariant models did not improve the accuracy; it may be that the size of the training data set is sufficient to overcome the lack of invariance.

While the accuracies obtained for top-1, −3 and −5 predictions are not sufficiently high for applications where the anticipation of all possible polymorphs is crucial, we believe that the accuracies offered by the best models from this work have value where CSP is used to quickly screen large areas of chemical space, either in a high-throughput manner^39^ or using guided searches of chemical space. For example, when used within an evolutionary algorithm for molecular discovery,^40^ we have recently demonstrated that the information from incomplete CSP landscapes can be effective in guiding methods to promising molecules. In these applications, there is a trade-off between completeness of the CSP landscape and lowering computational cost per molecule, which allows for assessing more candidate molecules. Also, we expect that there is room for improvement in space group prediction from molecular features. The similar accuracies obtained with two different machine learning models and different representations of the molecules within random forest and graph neural networks suggest that we are close to the accuracy limit in making such predictions. The accuracy is, of course, limited by the reality that some molecules form multiple polymorphs with different space groups.

## Supplementary Material



## Data Availability

Scripts used for training the random forest and graph neural network models can be found at github.com/hannahgittins/SG_ML_prediction. The data used in training of the models can be accessed at https://doi.org/10.5258/SOTON/D3912.

## Notes

List of features RF and GNN hyperparameters further RF results GNN training curves (PDF)
