# Supplementary material for: Can Machine Learning Predict the Space Group Preference of Organic Molecules?
Source: Cryst Growth Des. 2026 Apr 16;26(9):3318–26. doi: 10.1021/acs.cgd.5c01648 (PMC13154125; doi:10.1021/acs.cgd.5c01648)
Supplement: Supplementary file 1 [file cg5c01648_si_001.pdf]

Can machine learning predict the space group preference of organic molecules? - Supplementary Information

Hannah Gittins and Graeme M. Day

1 Random forest

1.1 Features

Category	Feature
General	Molecular weight
	Number of atoms
	Number of rings
	Number of aromatic rings
	Charge
Geometric	Molecular asphericity
	Eccentricity
	Internal shape factor
	Normalised principle moment ratio 1
	Normalised principle moment ratio 2
	First principal moment of inertia
	Second principal moment of inertia
	Third principal moment of inertia
	Radius of gyration
	Molecular sphericity index
	Deviation from plane of best fit
	Number of operations
	Symmetry operations
Chemical	Topological polar surface area
	Alcohol
	Amide
	Imine
	Nitro group
	Nitrile
	Amine
	Ether
	Aldehyde
	Halide
	Ketone
	Carboxylic acid
	Anhydride
	Ester
	Thiol
	Thiocarbonyl
	Thioether
	Sulfone
	Phosphoric acid

Table 1S: Features included in the training on the random forest model. All features have been normalised.

1.2 Hyperparameters

For the RF geometry optimised models, we undertook hyperparameter tuning using `Optuna` for the following hyperparameters and limited the search to:

- Number of decision trees - 10 to 1000 trees divided into steps of 10
- Max number of features used to train a tree - Log2 (log 2 of N features in training) and Sqrt (square root of N features)
- Maximum depth of the decision tree - 10-100 divided into steps of 10
- Minimum number of branches to split a node - 2 to 10
- Minimum number of samples required per leaf - 1 to 10
- Bootstrap (return features back into the pool) - True or False

After 100 trials, the best hyperparameters for the balanced model are:

- 820 decision trees,
- Max features = Log 2 of N features
- Minimum number of branches to split a node = 8
- Minimum number of samples required per leaf = 4
- Bootstraping = False

These hyperparameters were applied to both the geometry optimised unbalanced (RF_A) and balanced models.

1.3 Unimportant feature removal

The removal of unnecessary features on the geometry optimised balanced dataset was calculated and assumed to be the same for the geometry optimised unbalanced (RF_A) dataset to reduce computational cost. We classified useless features as features with low variance across themselves or high correlation with another feature (in case one feature is retained and the other is removed). The following features were classified as having a high correlation with another feature (with a threshold of r=±0.9):

- Anhydride
- Normalised principal moment ratio 1

A correlation map from `numpy` was used to find features that have a high correlation with another feature. For low-variance features, `VarianceThreshold` module from `sci-kit learn` with a threshold of 0.0001 (as all features were normalised between 0 and 1) which highlighted the following features:

Feature	All zero?
Thiol	N
Internal Shape Factor	N
First (smallest) principal moment of inertia	N
Second principal moment of inertia	N
Third (largest) principle moment of inertia	N
Radius of Gyration	N
Charge	Y
E	(all ones)
$C2$	Y
$C2'$	Y
$C2''$	Y
$C2_z$	Y

Continued on next page

Feature	All zero?
$C2_y$	Y
$C2_x$	Y
$C3$	Y
$C3^2$	Y
$C4$	Y
$C4^3$	Y
$C5$	Y
$C5^2$	Y
$C5^3$	Y
$C5^4$	Y
$C6$	Y
$C6^5$	Y
$C7$	Y
$C7^2$	Y
$C7^3$	Y
$C7^4$	Y
$C7^5$	Y
$C7^6$	Y
$C8$	Y
$C8^3$	Y
$C8^5$	Y
$C8^7$	Y
σh	Y
σv	Y
σd	Y
σv_{xz}	Y
$\sigma' v_{yz}$	Y
σ_{xy}	Y
σ_{xz}	Y
σ_{yz}	Y
i	Y
$S3$	Y
$S3^5$	Y
$S4$	Y
$S4^3$	Y
$S5$	Y
$S5^3$	Y
$S5^7$	Y
$S5^9$	Y
$S6$	Y
$S6^5$	Y
$S8$	Y
$S8^3$	Y
$S8^5$	Y
$S8^7$	Y
$S10$	Y
$S10^3$	Y
$S12$	Y

Table 2S: Features with low variance across themselves (according to the threshold of 0.0001) for the balanced, optimised, and no unique polymorphs training dataset. Features with all zeros (or all ones) were flagged in the right column.

1.4 Precision and recall

Space group	Top 1 /%		Top 3 /%		Top 5 /%		Top 10 /%	
	Precision	Recall	Precision	Recall	Precision	Recall	Precision	Recall
14	42.11	96.53	82.77	100	94.08	100	99.05	100
2	45.95	1.72	69.63	93.31	87.76	100	97.92	100
19	39.45	23.38	67.86	98.81	87.09	100	97.79	100
4	64.29	1.70	77.75	37.5	82.27	92.61	96.17	100
61	0	0	56.00	5.38	68.08	78.08	92.53	100
15	0	0	57.89	5.00	63.71	56.82	91.29	100
33	0	0	0	0	0	0	83.40	100
9	0	0	0	0	0	0	75.98	98.41
29	0	0	0	0	0	0	76.92	93.22
5	0	0	0	0	74.07	19.51	71.84	90.24
1	0	0	0	0	0	0	74.71	54.17
60	0	0	0	0	0	0	70.59	20.69
7	0	0	0	0	0	0	73.39	33.33
18	0	0	0	0	0	0	76.92	33.33
A	0	0	0	0	0	0	0	0
148	0	0	0	0	0	0	86.96	28.57
B	0	0	0	0	0	0	0	0
78	0	0	0	0	0	0	100	25.00
144	0	0	0	0	0	0	90.91	25.00
C	0	0	0	0	0	0	0	0

Table 3S: Precision and recall for the unbalanced model RF_A using the hold-out test set, arranged by space group frequency in the dataset. A = space groups 43, 56 and 88, B = space groups 13, 96, 92, 76 and 145 and C = space groups 169, 170, 20, 86, 45, 114, 41, 146, 82, 152, 154, 161, 110, 79, 106, 80, 173, 147, 85, 77, 54, 34, 52, 178, 37, 179, 171, 73, 159, 172, 94, 32, 70, 30, 81, 68, 3, 122, 153, 167, 27, 23, 91, 17, 130, 104, 142, 182, 118, 117, 21, 22, 36, 103, 98, 95, 120, 163, 158, 197 and 198.

It is worth noting that the following space groups are in the training set but not the test set for the unbalanced models: 130, 3, 142, 17, 147, 21, 22, 23, 153, 27, 158, 30, 32, 34, 163, 36, 37, 167, 41, 172, 173, 178, 179, 54, 182, 68, 197, 70, 198, 73, 77, 79, 81, 91, 94, 95, 98, 103, 104, 117, 118, 120, 122. This is likely due to these space groups making up a small proportion of the dataset, as a result, it is not surprising that the space groups would have poor precision and recall given that the model has not been tested for them.

Space group	Top 1 /%		Top 3 /%		Top 5 /%		Top 10 /%	
	Precision	Recall	Precision	Recall	Precision	Recall	Precision	Recall
1	20.51	32.00	36.36	48.00	54.05	64.00	72.29	72.00
2	9.52	16.00	20.39	28.00	32.89	40.00	57.55	64.00
4	7.14	4.00	22.73	20.00	30.00	24.00	58.82	52.00
5	11.11	16.00	35.29	48.00	49.67	60.00	69.77	72.00
7	25.00	24.00	32.26	40.00	35.46	40.00	65.84	64.00
9	14.29	4.00	39.13	24.00	43.01	32.00	55.81	48.00
13	11.54	12.00	35.00	28.00	43.69	36.00	67.96	56.00
14	4.76	4.00	19.35	16.00	18.87	16.00	59.52	60.00
15	9.52	8.00	17.39	16.00	23.15	20.00	52.42	52.00
18	9.09	8.00	17.39	16.00	37.41	44.00	66.42	72.00
19	9.09	4.00	20.93	12.00	26.60	20.00	57.78	52.00
29	9.09	12.00	31.58	40.00	44.22	52.00	67.86	76.00
33	7.69	8.00	16.90	16.00	22.52	20.00	60.47	52.00
43	25.00	16.00	27.27	20.00	31.58	24.00	58.54	48.00
56	7.14	4.00	17.91	16.00	32.37	36.00	59.93	64.00
60	0.00	0.00	9.68	4.00	20.83	12.00	59.63	52.00
61	4.00	4.00	11.11	8.00	20.41	16.00	57.69	60.00
76	0.00	0.00	3.90	4.00	22.73	24.00	55.35	60.00
78	4.35	8.00	13.04	16.00	27.03	32.00	56.82	60.00
88	17.24	20.00	27.59	32.00	41.35	44.00	66.67	76.00
92	3.85	4.00	16.44	16.00	33.98	28.00	67.51	64.00
96	18.75	12.00	16.22	16.00	38.71	48.00	65.07	76.00
144	5.00	8.00	20.00	28.00	32.05	40.00	63.83	72.00
145	6.45	8.00	31.73	44.00	41.40	52.00	65.04	64.00
148	12.50	16.00	34.62	36.00	40.44	44.00	64.89	68.00

Table 4S: Precision and recall for the balanced RF model.

1.5 RF model trained on geometries from the crystal

In the testing processes of this model, we trained a handful of RF models with geometries taken straight from the crystal structure. This was to test that the model could learn from the data before geometry optimisation. For these models, we carried out hyperparameter tuning in the same way as discussed - hyperparameter tuning with the balanced dataset and assuming the same for the unbalanced.

We obtained the following results:

	Accuracy from known statistical distribution /%	Accuracy /%	Δ /%
Top 1	40.22	44.58	4.36
Top 3	73.60	77.51	3.91
Top 5	87.11	88.43	1.32
Top 10	95.50	95.98	0.48

Table 5S: Accuracy of the unbalanced model trained with the geometries from straight from the crystal (RF_B). The irrelevant features were removed before training.

	Random accuracy /%	Accuracy /%	Δ /%
Top 1	4.01	9.44	5.43
Top 3	11.98	23.04	11.06
Top 5	20.02	33.92	13.90
Top 10	40.00	59.84	19.84

Table 6S: Accuracy of the 25-space group balanced RF model trained with the geometries from straight from the crystal. The irrelevant features were removed before training.

	Random accuracy /%	Accuracy /%	Δ /%
Top 1	9.98	18.69	8.71
Top 3	29.97	47.86	17.89
Top 5	49.96	67.63	17.67

Table 7S: Accuracy of the 10-space group balanced RF model trained with the geometries from straight from the crystal. The irrelevant features were removed before training.

For both the unbalanced and the balanced model, the accuracy is greater than the accuracy for the models trained with the optimised geometries (RF_A). This is expected as the model is learning the mapping between the molecular geometry in the crystal and the space group.

1.6 RF model trained with all polymorphs

In addition to the model trained on the geometries of the crystal, another model was trained with all polymorphs with unique space groups. This surprisingly barely caused the dataset to increase in size. The dataset went through all the same processes: geometry optimisation, hyperparameter tuning, useless feature removal, as well as creating a balanced set from the unbalanced set.

	Accuracy from known statistical distribution /%	Accuracy /%	Δ /%
Top 1	38.57	43.39	4.82
Top 3	72.66	75.97	3.31
Top 5	86.60	87.65	1.05
Top 10	95.61	94.95	-0.66

Table 8S: Accuracy of the unbalanced model with all polymorphs (RF_C). The irrelevant features were removed before training.

	Random accuracy /%	Accuracy /%	Δ /%
Top 1	4.00	10.56	6.56
Top 3	12.01	25.44	13.43
Top 5	20.01	36.16	16.15
Top 10	39.99	58.56	18.57

Table 9S: Accuracy of the 25-space group balanced model with all polymorphs. The irrelevant features were removed before training.

1.7 10-fold Cross Validation plots

	Accuracy from known statistical distribution /%	Chemical features only accuracy/%	Geometry features only accuracy/%	All features accuracy/%
Top 1	39.28 ± 0.41	40.82 ± 0.41	40.39 ± 0.23	41.93 ± 0.38
Top 3	73.00 ± 0.22	74.31 ± 0.16	74.22 ± 0.30	75.15 ± 0.18
Top 5	86.74 ± 0.21	87.36 ± 0.10	87.63 ± 0.31	87.84 ± 0.17
Top 10	95.81 ± 0.13	96.08 ± 0.08	96.14 ± 0.11	96.29 ± 0.11

Table 10S: 10-fold cross-validation for Model RF_A unbalanced model, as shown in Figure 1

	Random accuracy /%	Chemical features only accuracy/%	Geometry features only accuracy/%	All features accuracy/%
Top 1	3.99 ± 0.08	6.89 ± 0.86	7.61 ± 0.55	8.75 ± 1.20
Top 3	12.01 ± 0.11	19.91 ± 1.26	19.58 ± 1.50	22.77 ± 1.53
Top 5	19.96 ± 0.18	31.13 ± 1.74	31.33 ± 1.48	35.56 ± 2.03
Top 10	39.95 ± 0.15	54.22 ± 1.19	57.40 ± 1.99	60.80 ± 1.91

Table 11S: 10-fold cross-validation for the 25-space group balanced RF model, as shown in Figure 2

	Random accuracy /%	Chemical features only accuracy/%	Geometry features only accuracy/%	All features accuracy/%
Top 1	9.98 ± 0.05	15.44 ± 0.87	15.97 ± 0.69	18.69 ± 0.50
Top 3	29.97 ± 0.10	41.85 ± 0.65	42.24 ± 0.99	47.86 ± 0.95
Top 5	49.96 ± 0.09	62.63 ± 0.79	62.78 ± 1.17	67.63 ± 0.64

Table 12S: 10-fold cross-validation for the 10-space group balanced RF model.

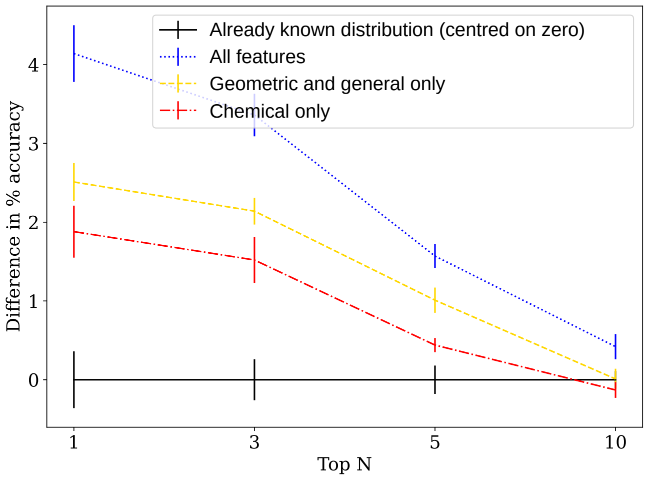

Figure 1S: Model RF_B: 10-fold cross-validation of the unbalanced model trained with the "ideal" case, geometries taken from the crystal, to 1 standard deviation. The already known distribution was centred at zero.

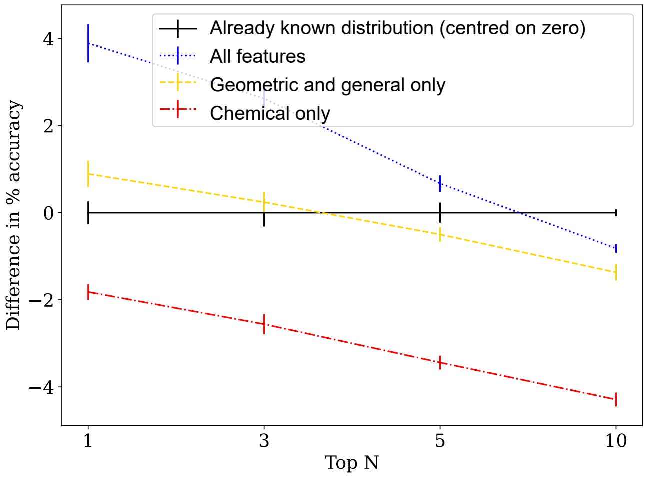

Figure 2S: Model RF_C: 10-fold cross-validation of the unbalanced model trained with all unique polymorphs to 1 standard deviation. The already known distribution was centred at zero.

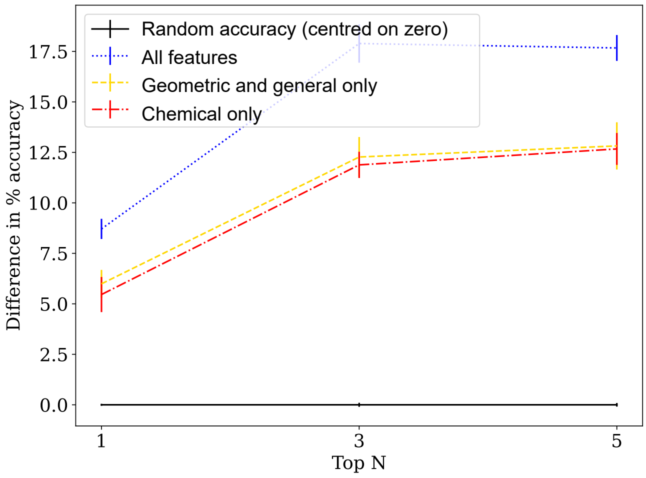

Figure 3S: 10-Space group balanced model, 10-fold cross-validation. The accuracy of random space group prediction is centred at zero.

1.8 Feature importance

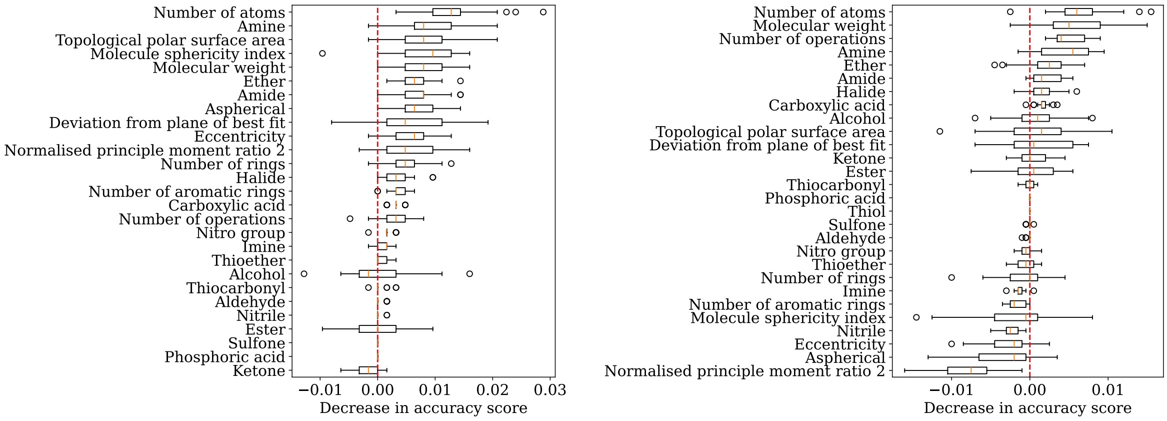

Figure 4S: Feature importance for the balanced test sets: (left) 25-space group balanced set; (right) 10-space group balanced set. Features have high importance if they reduce the RF accuracy when removed from the dataset.

1.9 Failed optimisations

List of CSD REFCODES where molecules failed optimisation, so are present in the dataset of molecular geometries taken from the CSD, but absent from the optimised molecule dataset: ALADAT, BUYQES, CECDAR, DOQQOQ, FIGKIP, FUMZOD, HALVOG, HALVUM, KEYTUE, KOTXIC, KUHTIS, LIH-WAB, NAJRIZ, NEMFEQ, PACWAT, PAMPIG, PUXDIV, QUFQIR, RAWTOY, RILNEI, ROJYAR, SOSZUW, SPIROC, TATNBZ, VUJREZ, WICBUF, XONNAQ, YIWJOD, YIWJUJ, YIWKEU, YO-JYEB, YOJYOL, ZEGMOQ

2 GNNs

2.1 Training loss plots

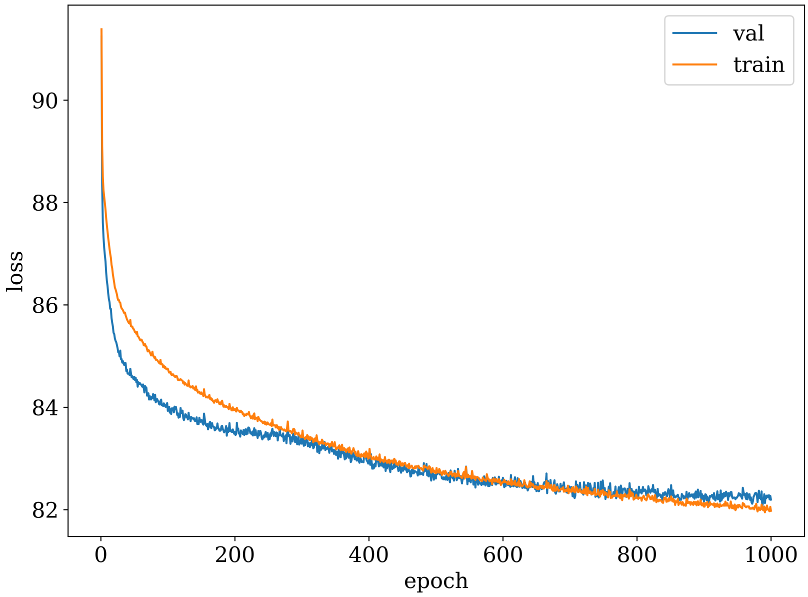

Figure 5S: Model GNN_1 epoch v. loss. By eye at around 900 epochs, the model starts to overfit (validation loss plateau, but training loss continues to dip, indicating overfitting). The best model based on validation loss is saved; therefore, the model around 700 epochs (before overfitting) will be taken forward.

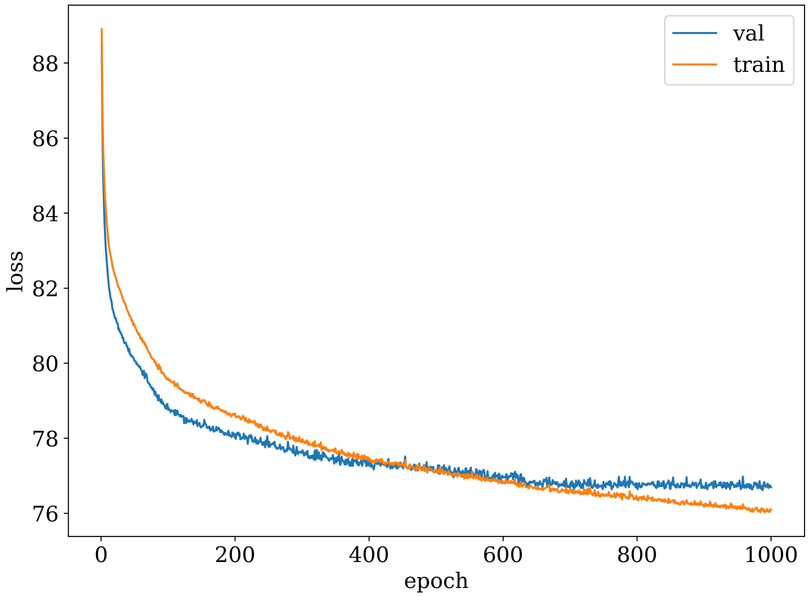

Figure 6S: Model GNN_2 epoch v. loss. By eye at around 700 epochs, the model starts to overfit (validation loss plateau, but training loss continues to dip, indicating overfitting). The best model based on validation loss is saved; therefore, the model around 900 epochs (before overfitting) will be taken forward.

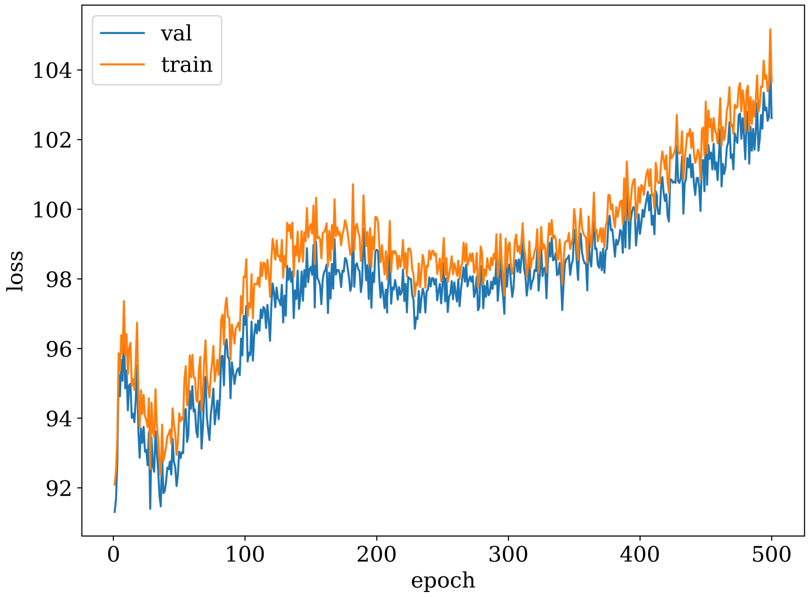

Figure 7S: Model GNN_3 epoch v. loss. For the augmented data model, the loss appeared to get out of control after around 40 epochs. We believe this occurred due to the model not being able to learn a pattern because the coordinates were changing. The best model was saved and taken for testing.

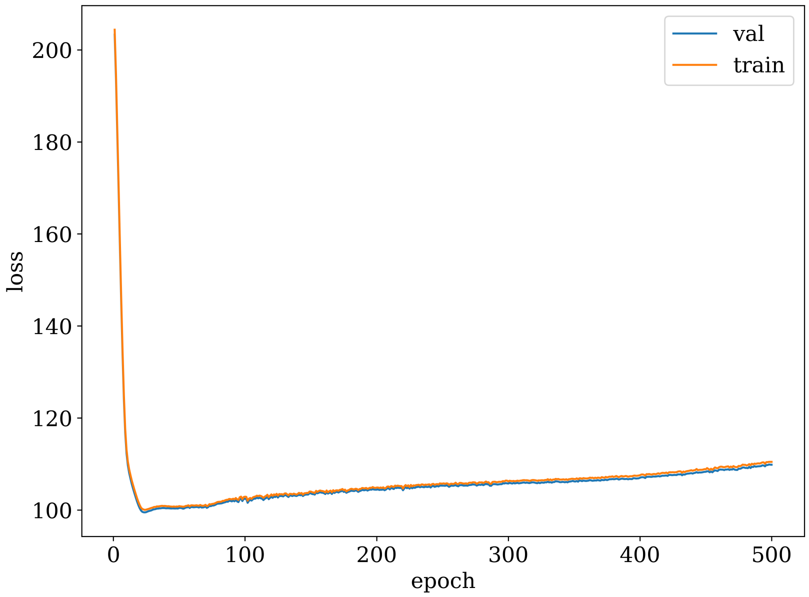

Figure 8S: Model GNN_4 epoch v. loss. With the EGNN model it struggles to learn from the data, and the loss increases rather than decreases as expected. It is not clear why this occurs, but the best model (best validation loss) is the one that is taken forward.
